# A Compendium on Perinatal Autopsy in Neonats

**DOI:** 10.7759/cureus.33878

**Published:** 2023-01-17

**Authors:** Joshi Abishake, Sudhir Ninave, Akshunna Keerti

**Affiliations:** 1 Medicine and Surgery, Jawaharlal Nehru Medical College, Datta Meghe Institute of Medical Sciences, Wardha, IND; 2 Forensic Medicine, Jawaharlal Nehru Medical College, Datta Meghe Institute of Medical Sciences, Wardha, IND

**Keywords:** fetal anomalies, karyotyping, fetal abnormality, postmortem, perinatal autopsy

## Abstract

Professionals who work in perinatal care must understand the advantages and disadvantages of perinatal autopsy since they are an essential tool for determining fetal and neonatal mortality. Perinatal is the period five months before one month after birth, while prenatal is before birth. The traditional prenatal autopsy is still the gold standard for establishing the cause of death and providing an accurate report, notwithstanding the development of new technology. The ideal locations for a prenatal autopsy are tertiary institutes that offer these procedures. It emphasizes the need for systematic histopathologic sampling, rigorous record-keeping, technological adaptation, and wise laboratory test use. When a laboratory does a microbiologic examination with a focus on the genital tract and neonatal problems, it is very beneficial. Karyotyping needs to be selective and works best when there are many aberrations if resources are to be saved. A perinatal autopsy is insufficient without examining the placenta, and severe lesions should be distinguished from deformities and abnormalities brought on by fetal death. In addition to providing epidemiology teams and auditing committees with high-quality data, the pathologist's role in perinatal medicine also includes participating in the multidisciplinary management of fetal abnormalities identified during pregnancy, monitoring the patterns of iatrogenic disease, and aiding the perinatal grief management process. Investigations into complicated multiple pregnancies, hydrops, bone dysplasias, and unexpected intrauterine fetal deaths provide unique obstacles and diagnostic difficulties. There hasn't been any research that contrasts postmortem computed tomography with postmortem x-rays in pregnant women, as far as we know. Histological analysis of many perinatal autopsies revealed healthy developing tissues. Only a tiny percentage of histological abnormalities can be expected in fetal anomaly terminations. On prenatal imaging, many organ abnormalities are commonly anticipated. A thorough database search was done in Pubmed, Medline, and Scopus using the phrases "fetal abnormalities," "karyotyping," "fetal abnormality," "postmortem," and "perinatal autopsy."

## Introduction and background

The gold standard approach for confirming anomalies in terminated or aborted fetuses, deceased neonates, or babies is still perinatal autopsy [1]. As a result, this inquiry is crucial to the imagistic assessments' quality control. Numerous high-quality research has compared the fetal and neonatal suspected abnormalities during the fetal ultrasound scan with the results of autopsies since the early inception of the anomaly scan [2]. To give helpful information for the parents counseling who are carrying deformed children, we reviewed the literature on the relationship between prenatal/perinatal abnormalities and fetal or neonatal autopsy [3]. 

In underdeveloped nations like India, congenital abnormalities account for 10-15% of perinatal mortality [4], and they continue to be one of the least emphasized areas of disease monitoring in India compared to infectious and certain chronic diseases [5]. In India, low birth weight, preterm, sepsis, and infections are still the leading causes of newborn mortality, in contrast to industrialized nations, where congenital abnormalities are the predominant cause of infant mortality [6]. As a result, the issue of genetic abnormalities in India has not received much attention [7]. 

The incidence and precision of prenatal ultrasound screening for fetal malformations have rapidly increased [8] due to the technical progress of ultrasound technology and the growth of obstetricians' and sonographers' prenatal diagnostic training during the past several decades [9]. Postmortem cross-sectional imaging allows the entire body to be examined without risking physical injury [10]. The patient's size makes it possible to perform volumetric reconstructions using postmortem computed tomography (CT) and magnetic resonance imaging (MRI), which promotes areas that can be virtually examined without the use of invasive procedures and documentation that is presented in a jury-friendly manner as opposed to conventional autopsy photos [11]. 

## Review

Methodology

To discover and evaluate the publications that assessed the consistency between fetal or neonatal autopsy and perinatal diagnosis of fetal malformations, we searched PubMed, Medline, and Scopus databases. "Fetal abnormalities," "karyotyping," "fetal abnormality," "postmortem," and "perinatal autopsy" were the search terms. We examined several articles and discovered that the various inclusion criteria of this research make it difficult to connect the findings of all of them. Variable neonatal fatalities, therapeutic abortions, and fetuses with intrauterine deaths or chromosomal abnormalities were all included in the research's design. While most studies examine the fetus, some focus solely on a particular fetal system, such as the cardiovascular, central nervous, or urinary systems. Another significant drawback of the literature that is currently available relates to the proper investigation of the accuracy of anomaly scans: almost all studies are retrospective; the autopsy rate in terminated pregnancies or stillbirths is typically reported at less than 75% - as advised in specialty guidelines, and the majority of papers have only included termination of pregnancy (TOP) due to anomalies picked up by prenatal ultrasound.

Changes occurring after death

Modifications that occur in a body after death, whether in utero or during the time between death and autopsy, produce traits that are thought to be typical postmortem changes [12]. On direct visual inspection, pediatric pathologists can often identify most of them, but further characterizing their imaging correlates is necessary [13].

The primary process that follows death is known as autolysis, which denotes the cellular breakdown of human tissues, likely fueled by enzymes. This process causes changes in tissue shape and permeability and fluid redistribution across body compartments. The imaging correlations of this are frequently observed. However, the rate at which such changes occur is not well understood and likely depends on several variables, including antemortem circumstances, age, mode of death, location of death, conditions for body storage, temperature, and a host of other factors [14]. Putrification also occurs concurrently with autolysis, secondary to bacterial colonization, which may cause further tissue deterioration and gas generation. Additional considerations, such as the impact of scavengers and insect activity, are uncommon in standard pediatric treatment and are not taken into account here. On these general changes, approximate estimations of the time of death are frequently predicated [15]. Unfortunately, in-utero mortality with a time of retention before delivery, in which there is softening of the fetus within the fluid (in this example, amniotic fluid), is likely to worsen fetal postmortem alterations [16]. This process is known as maceration. The pathologist can quickly identify maceration-related alterations on an external examination as skin sliding, skin discoloration, and discoloration of the umbilical cord, followed by more widespread discoloration and liquification of the cranial contents with separation of the sutures. Mummification results from persistent intrauterine retention after death [17]. Table 1 below shows physiochemical changes occurring in the body following death.

**Table 1:** Physiochemical changes occurring in body following death This is a self-made table by the author.

Physiochemical changes occurring in body following death:-	
Algor Mortis	Body temperature cool to the environment after death.
Livor Mortis	Purplish- blue discoloration after death due to setting of blood by gravitational forces within dilated, toneless capillaries of the skin.
Rigor Mortis	Stiffness after death.
Decomposition	Putrefaction and autolysis.

Purpose of perinatal autopsy

Finding the person's cause of death, manner of death, mode of death, and state of health before death are the key objectives of an autopsy. They are identifying if any medical diagnosis and treatments were suitable before death is also included. The prenatal autopsy can provide critical information to the family, the clinician, and society [18]. Parents desire to learn the cause and method of their child's death; therefore, they consent to a postmortem examination [19]. It is relieving for the parents to know that whatever went wrong did not happen because they did something wrong and that there was nothing they could have done to avoid it. When a pregnancy terminates due to anomalies, the postmortem, which serves as an audit tool for diagnosis and diagnostic procedures, can confirm, change, or reject a prenatal diagnosis [20]. The information gleaned from the autopsy can help the parents and the clinicians plan for subsequent pregnancies and help the clinicians advise the parents about potential recurrence risks, regardless of whether it was a stillbirth, miscarriage, or pregnancy termination due to fetal malformations [21]. In situations of newborn deaths, the perinatal autopsy can provide neonatologists with details on the precision of their diagnosis and any missing issues [22]. It could also include information on the effects of various drugs and medical treatments on tissues and organs. To properly care for mothers and their babies, many health professionals rely heavily on autopsy as a teaching tool. This group includes pathologists, pediatricians, neonatologists, obstetricians, midwives, nurses, and grief counselors. The prenatal autopsy can provide information that can improve public health [23]. Accurate cause-of-death data are needed to plan for national perinatal mortality statistics and health services [24].

Clinical Evidence

Any postmortem examination must have sufficient clinical information [25]. This is so that the pathologist may choose the optimum analysis technique, such as the manner of evisceration and evacuation of the brain and what additional studies are required. Additionally, understanding what research has been done before, during, or after delivery would assist avoid needless duplication. Having the mother's (and baby's) clinical notes would be excellent [26]. This might not be realistic, given how frequently hospitals send infants for postmortem investigation [27]. An appropriately filled-out autopsy request form is an option. Such a document should include the mother's prior medical history, including any ailments that could impact the pregnancy's outcome, such as diabetes and persistent hypertension [28]. It is crucial to account for previous pregnancies and how they turned out. The gestation based on the mother's most recent period for the index pregnancy should be reported, as well as any adjustments made using ultrasound imaging. Pre-eclampsia, gestational diabetes, pyrexia, and antepartum hemorrhage should all be pregnancy problems. Pregnancy-related investigations and their findings are crucial [29]. A copy of any abnormal scan findings is necessary if the mother's clinical records are unavailable, especially if the pregnancy was terminated owing to fetal malformations. Birth weight, delivery manner, and delivery time and date should all be listed on the form [30]. For live births, the newborn's health at birth, postpartum development, including any medical interventions, and the clinical reason for death ought to be reported. Table 2 below shows the causes of prenatal, neonatal and postnatal.

**Table 2:** Causes of Prenatal, Neonatal and Postnatal Rh - Rhesus factor [31]

Prenatal	Neonatal	Postnatal
Wanted/unwanted, attempted or threatened abortion	Prolonged labour duration	Infections
Rh incompatibility	Respiratory distress	Jaundice
Diabetes	Birth Weight	Convulsions
Hypertension	Respiratory distress	Injury
Trauma	Feeding problems	Failure to thrive
Nutrition	Colour of the baby	Nutritional disorders

Equipment utilized

A professionally equipped morgue with enough light and appropriately sized tables and chairs is necessary for the prenatal postmortem [31]. For a perinatal pathologist, high-tech photographic tools (cameras and stands) that enable capturing tiny fetuses and organs are crucial. Without this, a recent, compact camera with an excellent macro feature ought to do. Also necessary is radiographic equipment, typically in the form of a Faxitron [32]. Accurate weighing scales are needed for fetuses and newborns being autopsied. A digital display-equipped electronic balance is best to record weights to the closest 0.1 g [33]. A fixed board with a fixed end and adjustable foot, or a metric ruler with calipers, can be used to measure the lengths of the body and feet. Circumference measurements can be made with a string or a piece of tape [34]. Blades, scissors, forceps, and probes of various sizes are necessary for dissecting young infants and fetuses. Brushes and bowls of the proper size help handle little, delicate brains. Examining tiny fetuses and organs may require a mounted magnifier or a dissecting microscope.

Procedure and assessment during perinatal autopsy

The body is sent to a hospital, municipal mortuary, or medical examiner's office in a body bag or evidence sheet. A brand-new body bag is utilized for each body to guarantee that the pack only contains evidence related to that body. Evidence sheets serve as a substitute for moving the body. A sterile sheet that covers the body while it is transferred is known as an evidence sheet. The exterior and internal examinations are the two components of the physical inspection of the body [35]. These are usually supplemented by toxicology, biochemical tests, genetic studies, or molecular autopsies, which frequently help the pathologist identify the cause or causes of death. A "tip-to-toe" check is performed during the external examination to search for anomalies in the infant or fetus. The pathologist can determine the degree of maceration and get information about the time of death by evaluating skin color, the presence or absence of skin slippage, and the extent of skin slippage if present [36]. The baby's skin tone can also gauge or confirm the baby's gestation since extremely preterm newborns have bright pink skin, while post-term babies may have dry, wrinkled skin. The pathologist can advise the doctor or the laboratory to perform a Kleihauer test (if not already done) while the chance is still there. Pale skin can suggest fetal anemia, and in near-term or term newborns, it raises the likelihood of feto-maternal hemorrhage. Other characteristics that can be evaluated in newborn fatalities include bruises, petechial hemorrhages, edema, and jaundice [37]. Even with miscarriages and stillbirths, any injuries associated with delivery, such as abdominal wall deformities, should be carefully noted to distinguish them from natural anomalies. Parents frequently ask to view pictures of their infants, and it is crucial to document any birth-related injuries to avoid future grievances against the pathologist or the mortuary personnel. The coroner or medical examiner may also look into cases of probable birth trauma in intrapartum or neonatal fatalities [38]. Therefore, the need for meticulous and thorough injury documentation cannot be overstated. Sites of venepuncture drains, catheters, and surgical incisions should all be recorded in cases of newborn mortality. Until the location of the tip is examined internally, all drainage tubes, central lines, and umbilical arterial and venous catheters should be left in place [39]. The internal examination entails dissecting the body's internal organs and looking for signs of trauma or other clues to the cause of death. In the prenatal autopsy, a Faxitron whole-body radiograph is a helpful inquiry. The fundamental criteria are lateral and anterior-posterior views. Additional photos of specific areas, such as the chest or limbs, might be acquired as needed. The examination of skeletal dysplasia requires an excellent radiograph [40]. Additionally, it can be utilized to determine soft tissue calcification and gauge gestational age. In certain hospitals, postmortem MRI is becoming a more common complement to postmortem assessment, remarkably when the pregnancy was terminated due to brain abnormalities. MRI is beneficial in examining the mushy brain before the skull is opened, particularly in macerated fetuses [41]. Anterior, posterior, and lateral views are a bare minimum. Collecting further photographs of dysmorphic features and any other abnormalities found during external and interior investigations may be necessary. Any irregularity may be more accurately and reproducibly documented through photography than it can through textual explanation [42]. A second opinion on complex situations may be required, and clinical geneticists and pathologists may benefit from clear images. They can also be utilized for training and instructional reasons, as well as to illustrate the numerous anomalies at perinatal mortality meetings. On request, some facilities offer family photos.

Discussion

Particularly in this era of notable advancements in prenatal diagnosis, a fetal autopsy should be routinely indicated in the treatment of the identified anomalies, as it gives crucial information in about 25% of cases or even changes the prenatal diagnosis in at least 5% of cases [43]. The alarming decline in autopsy rates can be attributed to several factors, including the centralization of pathology services, adjustments in clinicians' perceptions of the value of the investigation (primarily as a result of improvements in diagnostic imaging), poor counseling provided by non-experts in fetal medicine, or the absence of a pathologist from the counseling team. Parents choose not to consent to a typical, invasive autopsy due to these conditions [44]. Since it required more and more expertise over time, perinatal pathology developed into a specialized field of general pathology. We should consider autopsies' role in teaching and research and the audit function for prenatal diagnostic and treatment approaches. However, the essential aspect of the perinatal autopsy is to confirm and complete the diagnosis of fetal abnormalities, including characteristics not visible during pregnancy, and to fine-tune the preliminary diagnosis, which may call for histological, genetic, or X-ray examination [45]. Tissue samples can also be kept for future microscopic, genetic, and biochemical research if these investigations were not conducted during pregnancy. Any post-mortem assessment must have sufficient clinical information. This is because the pathologist may choose the optimum examination technique, such as the manner of evisceration and evacuation of the brain and what additional studies are required. Additionally, understanding what research has been done before, during, or after delivery would assist in avoiding needless duplication.

## Conclusions

The prenatal imagistic assessment of the pregnancy's information has become crucial to regular prenatal treatment, and the ability to detect developmental defects has considerably increased. However, to confirm or enhance the prenatal imagistic diagnosis, parents should be persuaded to adopt the gold standard pathological examination and correct perinatal autopsy technique, which should be carried out in all therapeutic terminations of pregnancy or stillbirths. The strong collaboration between pathologists and ultra-sonographers is beneficial for parental counseling for upcoming pregnancies and the advancement of sonographic anomaly diagnostics and perinatal pathology. A crucial component of the neonatal autopsy should be the collection of tissue samples for further examination. Congenital abnormalities are a significant cause of perinatal mortality, and fetal autopsy dramatically aids in detecting intrauterine fetal death. The capacity to detect developmental problems has significantly improved, and routine prenatal care now mainly relies on the knowledge obtained through prenatal imagistic pregnancy assessment. However, parents should be urged to use the gold-standard pathological examination and proper perinatal autopsy procedure that should be performed in all therapeutic terminations of pregnancy or stillbirths to confirm or strengthen the prenatal imagistic diagnosis. Pathologists and ultrasonographers work closely together to progress sonographic anomaly diagnosis, perinatal pathology, and parental counseling for future pregnancies. Collecting tissue samples for further investigation should be a vital part of the newborn autopsy.
